# Four-dimensional lipidomics profiling in X-linked adrenoleukodystrophy using trapped ion mobility mass spectrometry

**DOI:** 10.1016/j.jlr.2024.100567

**Published:** 2024-05-23

**Authors:** Yorrick R.J. Jaspers, Sven W. Meyer, Mia L. Pras-Raves, Inge M.E. Dijkstra, Eric J.M. Wever, Adrie D. Dane, Jan-Bert van Klinken, Gajja S. Salomons, Riekelt H. Houtkooper, Marc Engelen, Stephan Kemp, Michel Van Weeghel, Frédéric M. Vaz

**Affiliations:** 1 Laboratory Genetic Metabolic Diseases, Department of Laboratory Medicine, Amsterdam UMC location University of Amsterdam, Amsterdam, The Netherlands; 2 Amsterdam Gastroenterology Endocrinology Metabolism Institute, Amsterdam, The Netherlands; 3 Amsterdam Neuroscience institute, Amsterdam, The Netherlands; 4 Bruker Daltonics GmbH & Co. KG, Bremen, Germany; 5 Bioinformatics Laboratory, Department of Epidemiology and Data Science, Amsterdam Public Health Research Institute, Amsterdam UMC location University of Amsterdam, Amsterdam, The Netherlands; 6 Core Facility Metabolomics, Amsterdam UMC location University of Amsterdam, Amsterdam, The Netherlands; 7 Department of Pediatrics, Emma Children's Hospital, Amsterdam UMC location University of Amsterdam, Amsterdam, The Netherlands; 8 Emma Center for Personalized Medicine, Amsterdam UMC location University of Amsterdam, Amsterdam, The Netherlands; 9 Department of Pediatric Neurology, Amsterdam Leukodystrophy Center, Emma Children's Hospital, Amsterdam UMC location University of Amsterdam, Amsterdam, The Netherlands

**Keywords:** 4D-Lipidomics, parallel accumulation serial fragmentation, very-long-chain fatty acids, trapped ion mobility spectrometry, adrenoleukodystrophy

## Abstract

Lipids play pivotal roles in an extensive range of metabolic and physiological processes. In recent years, the convergence of trapped ion mobility spectrometry and MS has enabled 4D-lipidomics, a highly promising technology for comprehensive lipid analysis. 4D-lipidomics assesses lipid annotations across four distinct dimensions—retention time, collisional cross section, m/z (mass-to-charge ratio), and MS/MS spectra—providing a heightened level of confidence in lipid annotation. These advantages prove particularly valuable when investigating complex disorders involving lipid metabolism, such as adrenoleukodystrophy (ALD). ALD is characterized by the accumulation of very-long-chain fatty acids (VLCFAs) due to pathogenic variants in the *ABCD1* gene. A comprehensive 4D-lipidomics strategy of ALD fibroblasts demonstrated significant elevations of various lipids from multiple classes. This indicates that the changes observed in ALD are not confined to a single lipid class and likely impacts a broad spectrum of lipid-mediated physiological processes. Our findings highlight the incorporation of mainly saturated and monounsaturated VLCFA variants into a range of lipid classes, encompassing phosphatidylcholines, triacylglycerols, and cholesterol esters. These include ultra-long-chain fatty acids with a length of up to thirty carbon atoms. Lipid species containing C26:0 and C26:1 were the most frequently detected VLCFA lipids in our study. Furthermore, we report a panel of 121 new candidate biomarkers in fibroblasts, exhibiting significant differentiation between controls and individuals with ALD. In summary, this study demonstrates the capabilities of a 4D-lipid profiling workflow in unraveling novel insights into the intricate lipid modifications associated with metabolic disorders like ALD.

## Graphical abstract

**Figure:**
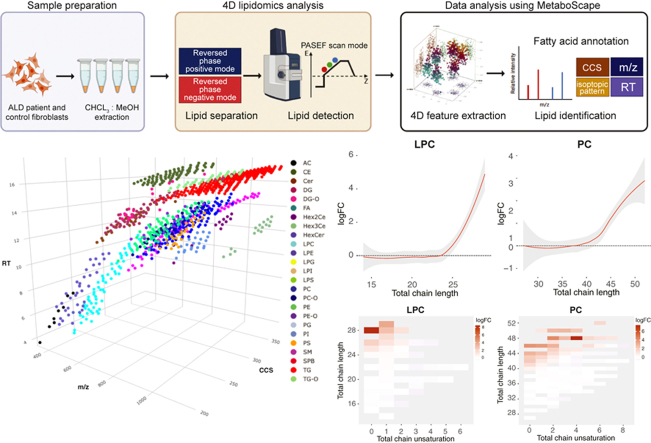

Lipids play essential roles in a wide range of metabolic and physiological processes, and dysregulation of lipid metabolism has been linked to various pathological conditions (1). Lipids are also of interest as diagnostic and predictive disease biomarkers, therapeutic agents, and targets for evaluating treatment response in clinical research (2). With the emergence of advanced high resolution MS-based profiling techniques, numerous novel lipid biomarkers linked to various diseases have been discovered in large sample cohorts (3, 4). This trend has sparked a growing interest in the clinical application of lipidomics to enhance disease diagnostics, monitoring, and treatment. However, identifying and quantifying lipids accurately in complex biological samples remains challenging due to the vast diversity and complexity of lipid molecular structures. New strategies that are able to enhance lipid analysis are therefore crucial toward more standardized MS-based lipidomics.

In recent years, ion mobility-enhanced MS has gained traction as a promising technology for lipidomics. By combining ion mobility with MS, this approach offers improved separation, enhanced peak capacity, and reduced chemical noise (5, 6). Moreover, the combination of LC separation with ion mobility-MS facilitates multidimensional separation and enhances the differentiation of lipid isomers, thereby enabling more comprehensive lipid profiling (7). Trapped ion mobility spectrometry (TIMS) is a relatively new and promising technique for ion mobility analysis (8, 9). In the TIMS analyzer, ions are transported by the push of a gas flow until they are positioned in an electrical field. Manipulation of the electrical field allows for the sequential release from the TIMS device thereby separating ions based on their collisional cross section (CCS) (10, 11). The resolution of the ion mobility separation is proportional to the user-defined ramp time of the electrical field. Utilizing a configuration that uses two TIMS cells, such as the timsTOF, provides significant advantages for increasing duty cycle and sensitivity. The first TIMS compartment is used for ion accumulation, while the second section is used for mobility separation thereby achieving a nearly 100% duty cycle (12). This enables parallel accumulation serial fragmentation (PASEF), a technique that has recently been applied in lipidomics (13, 14). PASEF synchronizes TIMS with MS/MS precursor selection in the quadrupole, allowing for rapid acquisition rates of MS/MS spectra. Due to the extra separation dimension that ion mobility provides, PASEF results in a less complex MS/MS spectra. In addition, a PASEF approach allows for the evaluation of the lipid annotations using four distinct dimensions: retention time (RT), CCS, m/z, and MS/MS spectra (15). This approach is referred to as 4D lipidomics and offers high-speed in-depth lipid analysis.

One of the areas where lipidomics has emerged as a powerful tool is in the study of metabolic disorders. By identifying and quantifying specific lipid species and their derivatives, lipidomics can offer valuable insights into the underlying mechanisms of disorders that are the result of disrupted lipid metabolism (16). One of the most common inherited metabolic disorders is X-linked adrenoleukodystrophy (ALD). ALD (OMIM: 300100) is a genetic neurometabolic disorder caused by pathogenic variants in the *ABCD1* gene with an incidence of 1 in 14,700 (17). The protein ALDP (ATP-binding cassette subfamily D member 1 protein), encoded by the *ABCD1* gene, is a peroxisomal membrane protein responsible for transporting very-long-chain fatty acids-CoA esters (VLCFAs; ≥C22:0) into peroxisomes. When ALDP is absent or dysfunctional, there is a disruption in peroxisomal β-oxidation leading to VLCFA accumulation in nearly all body fluids and tissues. Clinical symptoms have been described in detail elsewhere and can include spinal cord disease, adrenal insufficiency, and lesions within the white matter of the brain (referred to as cerebral ALD) (18). Prediction of clinical outcome in a particular individual is currently unpredictable and can be variable even within the same family (19).

Several studies have consistently demonstrated elevated levels of VLCFA in ALD across a variety of lipid extracts from different origins (body fluids and tissues). These fractions include phosphatidylcholines (PCs), cholesterol esters (CEs), and triacylglycerols (TGs) (20, 21). The pathophysiology of ALD is most likely due to the accumulation of VLCFA in complex lipids. In fact, abnormal incorporation of VLCFA into lipid fractions has been shown to affect their function. For instance, studies have demonstrated that the inclusion of these “abnormal” VLCFA containing lipids into cell membranes leads to alterations in the membrane viscosity of erythrocytes. (22) Furthermore, research with artificial phospholipid vesicles has revealed that VLCFA lipid fractions interfere with both membrane structure and function (23). The characterization of VLCFA accumulation in ALD has so far predominantly relied on VLCFA analysis using GC-MS. (24) This method involves the release of VLCFA from their molecular origin by a hydrolysis step in the sample preparation, followed by determining the concentrations of C22:0, C24:0, and C26:0 fatty acids using GC-MS. While this approach is considered the gold standard in the diagnostic workup for ALD, any information about the biomolecular origin of the individual VLCFA is lost. In recent times, lysophosphatidylchloline (LPC) 26:0 has surfaced as a potent marker for ALD diagnosis and neonatal screening. It surpasses VLCFA analysis in diagnostic performance (25). While LPC 26:0 is reflective of VLCFA accumulation within the lipid compartment, it leaves the broader impact of VLCFA accumulation on the lipidome largely unexplored. This leads to a lack of understanding about the extent to which different variants of VLCFA are integrated into different segments of the lipidome.

In the present study, we used an in-depth 4D-lipidomics approach that leverages PASEF to comprehensively characterize the ALD lipidome. Through our analysis, we identify 1,155 lipids including various VLCFA containing species. Furthermore, we characterize the incorporation and distribution of VLCFA in different lipid classes. Finally, we report a panel of new biomarker candidates. Collectively, this study demonstrates the power of 4D-lipidomics to uncover novel insights into the lipid alterations occurring in this intricate metabolic disorder.

## Materials and Methods

### ALD individuals

Fibroblast samples were collected from 10 healthy controls, and 11 individuals with ALD from the biobank linked to the Dutch ALD cohort (26). The diagnosis of ALD was confirmed by *ABCD1* mutation analysis and elevated VLCFA (C26:0, C26:0/C22:0, and LPC 26:0 levels for all ALD individuals). Cerebral ALD was present in two individuals with ALD and adrenal insufficiency was present in eight individuals with ALD. Written informed consent was received from each individual. The study protocol was approved by the local Institutional Review Board (METC 2018-310). Control fibroblasts were obtained from male anonymous volunteers with written informed consent. Fibroblasts were cultured as described earlier (27). Briefly, fibroblasts were cultured using Ham’s F-10 Medium supplemented with 10% fetal bovine serum (Invitrogen, Carlsbad, CA), 25 mM of Hepes, 100 U/ml of penicillin, 100 μg/ml of streptomycin, and 250 μg/ml of amphotericin in a humidified atmosphere of 5% CO_2_ at 37°C.

### Lipid extraction

Lipid extraction was performed as previously described (27). Protein concentrations of fibroblast homogenates were determined using a bicinchoninic acid assay (28). In a 2 ml tube, fibroblasts homogenates (equivalent to 200 μg of protein) were mixed with a solution of internal standards for different lipid classes, including 0.1 nmol of cardiolipin (CL) CL 14:0_14:0_14:0_14:0, 2.0 nmol of phosphatidylcholine PC 14:0_14:0, 0.1 nmol of phosphatidylglycerol (PG) PG 14:0_14:0, 5.0 nmol of phosphatidylserine (PS) PS 14:0_14:0, 0.5 nmol of phosphatidylethanolamine (PE) PE 14:0_14:0, 0.5 nmol of phosphatidic acid (PA) PA 14:0_14;0, 2.125 nmol of sphingomyelin (SM) SM d18:1_12:0, 0.02 nmol of lysophosphatidylglycerol (LPG) LPG 14:0, 0.1 nmol of lysophosphatidylethanolamine (LPE) LPE 14:0, 0.5 nmol of LPC, LPC 14:0, 0.1 nmol of lysophosphatidic acid (LPA) LPA 14:0, 0.5 nmol of phosphatidylinositol (PI) PI 8:0_8:0, 0.5 nmol diglycerides (DGs) DG 14:0_14:0, 0.5 nmol triglycerides TG 14:0_14:0_14:0, 2.5 nmol cholesterol ester D_7_-CE 16:0, 0.125 nmol of sphingosine and ceramide mix (Avanti Polar Lipids) dissolved in 1:1 (v/v) methanol:chloroform. Next, 1.5 ml 1:1 (v/v) methanol:chloroform was added to each sample. The mixture was sonicated in a water bath (5 min) and centrifuged (4°C, 16,000g, 10 min). The supernatant was transferred to a 1.5 ml glass auto sampler vial and evaporated under a stream of nitrogen at 45°C. The dried lipids were reconstituted in 100 μl of 1:1 (v/v) methanol:chloroform.

### LCMS analysis

Chromatographic separation of lipids was done using a Thermo Fisher Scientific Ultimate 3,000 binary ultra-performance liquid chromatography. LC separation was done using a Waters HSS T3 column (150 × 2.1 mm, 1.8 μm particle size). The composition of the mobile phase A consisted of 4:6 (v/v) methanol:water and B 1:9 (v/v) methanol:isopropanol, both containing 0.1% formic acid and 10 mmol/l ammonia. The gradient started at 100% A going to 80% A at 1 min and 0% A at 16 min, 0% A for 16–20 min, 100% A at 20.1 min, and 100% A for 20.1–21 min. The column temperature was held constant at 60°C and a flow rate of 0.4 ml/min was used. After LC separation, lipids were detected using Bruker Daltonics timsTOF pro 2 mass spectrometer. Ionization was done using vacuum insulated probe heated electrospray ionization source ion source in positive and negative ionization mode in separate runs. The end plate offset was set to 500 V, and the capillary voltage was set to 4,500 V in positive mode and 3,600 V in negative mode. Nitrogen was used as the dry gas at a flow rate of 8 L/min and the nebulizer gas was set at 2.5 bar. The dry temperature was 230°C and the sheath gas temperature was 400°C, using a sheath gas flow of 4 L/min. PASEF scan mode was used covering a mass range of 100–1,350 m/z. Active exclusion was set to 0.1 min. The exclusion window was 0.010 m/z and 0.010 Vs/cm^2^. The acquisition cycle was 100 ms (with a mobility scan range of 0.55–1.90 Vs/cm^2^. The Ramp rate was 9.42 Hz and the number of PASEF ramps was set to 2 resulting in a cycle time of 0.32 s. The collision energy was set to a fixed value of 30 eV in positive mode and 40 eV negative mode.

### Data processing, bioinformatics, and statistics

Data processing and lipid annotation were done using MetaboScape 2023b (https://www.bruker.com/en/products-and-solutions/mass-spectrometry/ms-software/metaboscape.html). Mass and mobility recalibration was done using sodium formate and Agilent Tune mix, respectively, which were inserted in defined calibration segment of each measurement. Lipid identification was done using MetaboScape 2023b utilizing an in-house generated RT and m/z database, rule-based lipid annotation and the MS DIAL MS/MS library LipidBlast (https://systemsomicslab.github.io/compms/msdial/main.html, version 68). For lipid identification, the Lipidomics Standards Initiative guidelines were followed, using lipid class-specific fragments to determine the lipid species and molecular lipid species-specific fragments for the annotation of hydrocarbon chains. (29) The Lipidomics Standards Initiative reporting checklist is provided in the supplementary material. Annotations were evaluated based on deviations in accurate mass (<5 ppm), CCS (<5%), isotopic pattern score (<150), and MSMS score (>500). The reported lipid abundances were semiquantitative, calculated by dividing the analyte response by that of the corresponding internal standard. If no appropriate internal standard was present for a certain lipid class, their intensities were normalized against the intensity of PE 14:0_14:0. Lipid saturation and chain length plots were generated using the R package lipidr (https://www.lipidr.org) (30). We used a Welch’s *t* test to compare lipid species between groups. Significance was determined with a threshold of *P*<0.05. To account for multiple testing within lipid classes, we applied a Benjamini and Hochberg adjustment to the *P*-values.

## Results

### In-depth lipid profiling using PASEF

**Fig. 1:**
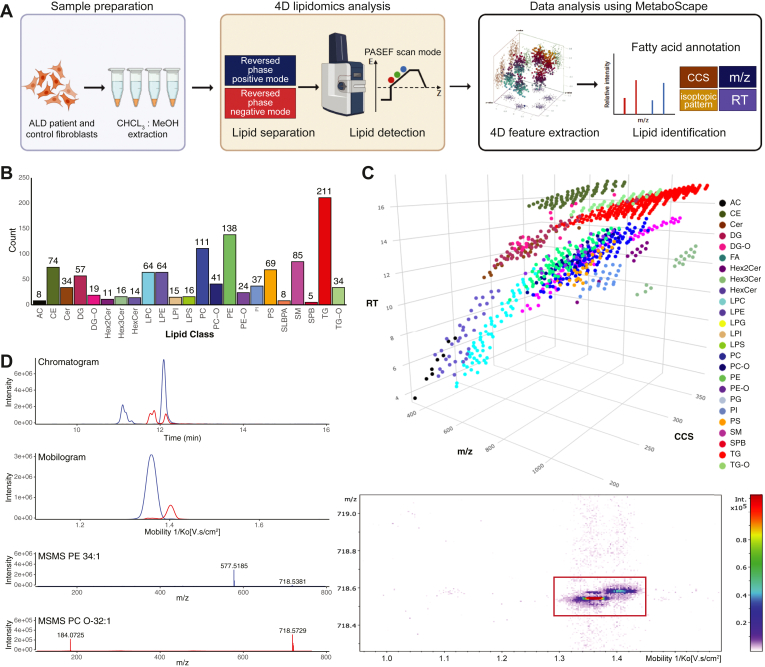
4D lipidomics profiling using PASEF (A) 4D lipidomics workflow. Samples are extracted using methanol:chloroform, followed by LC and TIMS mass spectrometry. Lipids are detected using PASEF which enables efficient precursor selection and MSMS acquisition. Features are identified using accurate mass, retention time, isotopic pattern, MSMS spectra, and CCS. B: Bar chart of detected lipid species per class. C: Three-dimensional representation (RT, m/z, CCS) of detected lipids. Different colors represent different lipid classes. D: Chromatogram of PE 34:1 (*blue*) and PC O-32:1 (*red*) show overlapping peaks in the LC dimension but are not overlapping in the ion mobility dimension. PASEF enables MSMS coverage for nearly every ion mobility resolved peak leading to noninterfering MSMS spectra of PE 34:1 and PC O-32:1. CCS, collisional cross section; PASEF, parallel accumulation serial fragmentation; PC, phosphatidylcholine; PE, phosphatidylethanolamine; RT, retention time; TIMS, trapped ion mobility spectrometry.

In this study, we used a 4D-lipidomics methodology to profile the lipidome of fibroblasts derived from individuals with ALD and healthy individuals (Fig. 1A). We used a simple methanol:chloroform lipid extraction protocol that can be applied to a wide variety of samples, such as cell lines, body fluids, and tissues. Lipids were effectively separated using a Waters HSS T3 column, which allowed us to separate lipids with a wide variety of polarities within a period of 21 min. Lipids were detected using PASEF, which facilitates the selection of multiple precursors for MS/MS acquisition within a single TIMS scan. Subsequent processing and analysis of the PASEF data using MetaboScape 2023b resulted in 9,182 features using positive ionization mode and 6,085 features using negative ionization mode. Notably, the high MS/MS acquisition rate of PASEF facilitated data availability for 85% (7885) of total features in positive ionization mode and 93% (5701) in negative ionization mode. We used MetaboScape 2023b for feature annotations, leveraging rule-based lipid annotation, the MS-DIAL LipidBlast spectral library, and an in-house generated RT and m/z library. Each annotation was evaluated on RT, mass deviation, isotopic pattern, MS/MS score, and CCS values. Phospholipids were detected using distinct adducts, including [M+H]^+^ or [M+NH_4_]^+^ in positive mode and [M-H]^-^ in negative mode. We primarily identified neutral lipids like DG and TG as [M+NH_4_]^+^ adducts in positive mode. This resulted in the identification of 1,155 unique lipids (Supplemental Table S1), encompassing a broad spectrum of classes (Fig. 1B). Among these 1,155 lipids, the spectral library successfully identified 1,094 lipids, whereas rule-based annotation identified 974, showcasing the substantial overlap between these two identification methods. A total of 154 lipids were detected exclusively by the LipidBlast spectral library and not by rule-based annotation. These mostly included lipids from classes such as TG, TG-O, and Hex3Cer. In contrast, 34 lipids were identified solely through rule-based annotation. These lipids belonged to a diverse range of classes, including CE and PE. Additionally, 27 lipids were exclusively identified by our in-house generated library. These lipids comprised acylcarnitines, which are not included in the rule-based tool or the LipidBlast library. While there is a significant overlap in lipid identification between the different methods, each one also provides unique contributions, demonstrating the value of using multiple strategies for comprehensive lipidomics analysis.

Figure 1C provides a 3D visualization that depicts all identified lipids, with colors representing their respective classes. In this representation, each lipid class clusters in distinct locations within the 3D space, highlighting the impact of variations in lipid composition and chemical structure. In our workflow, the elution of monoacyl lipids, primarily LPC and LPE, occurs within the first 10 min, with a CCS range of 200–270 Å^2^ (m/z 400–650). Between 12 and 15 min, DG (m/z 550–750, CCS 250–300 Å^2^) coelute with various glycerophospholipids, including PC, PE, and PG (m/z 650–950, CCS 270–330 Å^2^). Distinguishing between these lipids is achievable by both the m/z and CCS dimension. Beyond the 15-min mark, neutral lipids such as TG (m/z 900–1,100, CCS 300–360 Å^2^) and CE (m/z 600–860, CCS 270–330 Å^2^) elute. The PASEF acquisition mode not only strengthens the confidence in annotation but also facilitates the separation of coeluting masses within the ion mobility dimension. Figure 1D displays the overlapping extracted ion chromatograms of PE 34:1 (m/z 718.5381) and PC O-32:1 (m/z 718.5745) at 12.1 min. Despite concurrent chromatographic elution, these lipid species present distinct mobility values within the ion mobility dimension (Fig. 1D). One of the primary advantages of the PASEF acquisition mode is its potential to generate an MS/MS spectrum for nearly every individual ion mobility-resolved peak. This capability allowed for the effective isolation of the two precursor ions, leading to less complex MS/MS spectra.

### ALD induces in significant changes in the lipidome

**Fig. 2:**
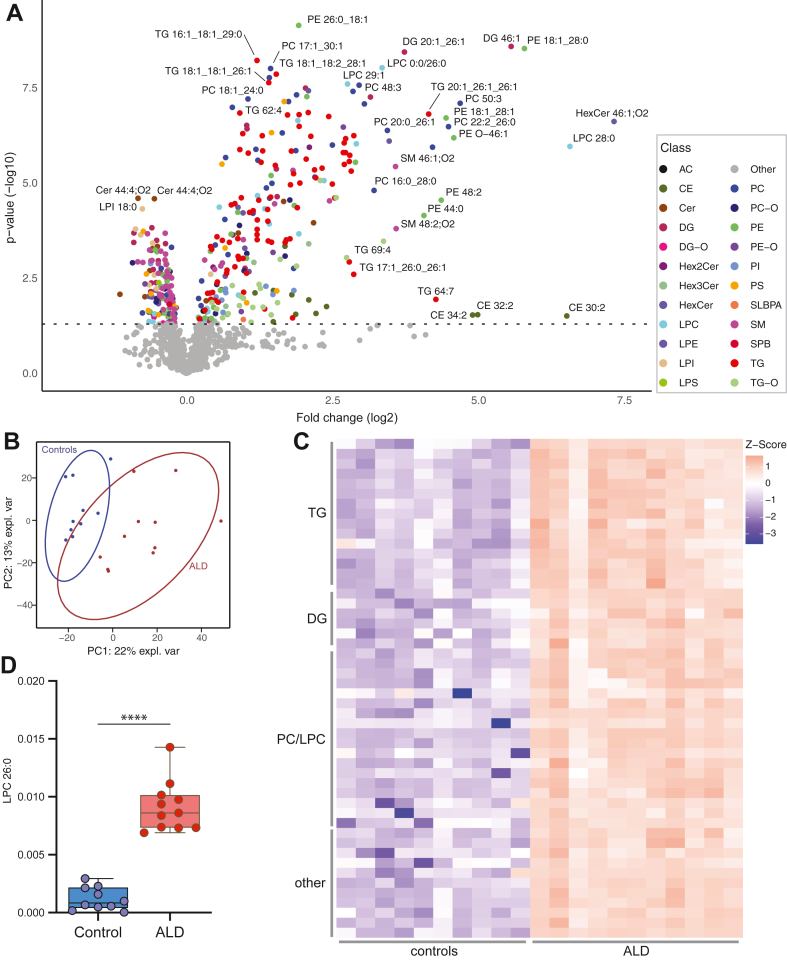
ALD induces in significant changes in the lipidome. A: Volcano plot of lipid levels. The vertical axis contains the *P*-value (−log10) from Welch t-tests between ALD and controls, and the horizontal axis the fold change (log2) between ALD and controls. Colored dots are lipids with a *P*-value of <0.05. Each color represents a different lipid class. B: PCA analysis shows distinct group of ALD and control fibroblasts. C: Heatmap of Z-scores of top 50 lipids based on *P*-value sorted on lipid class. D: Box plot of the newborn screening marker LPC 26:0 in ALD and control fibroblasts (∗∗∗∗*P* < 0.0001). ALD, adrenoleukodystrophy; LPC, lysophosphatidylchloline; PCA, principal component analysis.

ALD is characterized by a disruption in peroxisomal β-oxidation leading to accumulation of VLCFA and incorporation into complex lipids. To gain a deeper understanding of the impact of this disruption on the lipidome, we conducted a comparative analysis of lipid profiles obtained from fibroblasts of 11 male individuals with ALD and 10 healthy male controls. Using the 4D-lipidomics approach, we were able to uncover substantial differences between the lipid composition of ALD individuals and controls as illustrated by the volcano plot in Fig. 2A and further corroborated by the principal component analysis in Fig. 2B. After adjusting for the false discovery rate our analysis revealed 196 lipids significantly elevated (*P*<0.05, fold change >1.5), and 25 lipids significantly decreased in ALD individuals (Supplemental Table S2). Notably, the elevated lipids encompassed various classes of lipids, LPC, PC, DG, TG, CE, and others (Figure 2A, C). One of the most elevated lipids was 1-hexacosanoyl-sn-glycero-3-phosphocholine (commonly referred to as LPC 26:0), which is the marker that is used in ALD newborn screening. (31) LPC 26:0 showed a clear separation between ALD individuals and controls with no overlap (Fig. 2D). Decreased lipids encompassed a variety of lipid classes including PS, SM, DG, and ceramide. The majority of these lipids comprised shorter polyunsaturated variants, typically containing fewer than 22 carbon atoms for monoacyl lipids and less than 44 carbon atoms for diacyl lipids.

### A variety of VLCFA are incorporated in the ALD lipidome

**Fig. 3:**
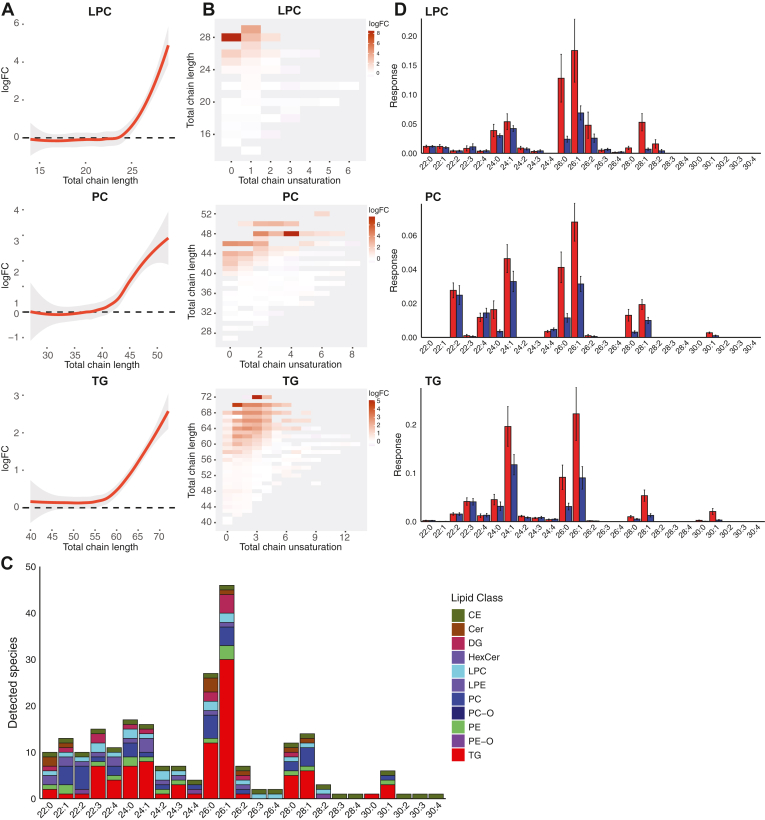
Characterization of VLCFA in the ALD lipidome. A: *Trend lines* illustrating the log fold change (logFC) and the total chain length of LPC, PC, and TG lipids when ALD is compared to controls. B: Heatmap illustrating the log fold change, total chain unsaturation and chain length for LPC, PC, and TG when ALD is compared to controls. C: Bar plot of amount of detected VLCFA containing species for LPC, PC, and TG. D: Sum of lipid species containing specific VLCFA variants for LPC, PC, and TG. ALD, adrenoleukodystrophy; LPC, lysophosphatidylchloline; PC, phosphatidylcholine; TG, triacylglycerol; VLCFA, very-long-chain fatty acid.

After identifying a substantial number of altered lipids in ALD, we proceeded to analyze the trends pertaining to the total acyl-chain lengths of these lipids. Figure 3A shows the total acyl-chain length and the fold change when ALD is compared against controls for LPC, PC, and TG. Total chain lengths of elevated lipids in ALD individuals were representative of VLCFA incorporation. For elevated lipids containing a single acyl chain, such as LPC and CE, the total acyl-chain length exceeded 20 carbon atoms, with a maximum of two double bonds (Fig. 3B). Lipids with two acyl chains, like PC and DG, had a total acyl-chain length greater than 40 carbon atoms, accommodating up to 6 double bonds. Furthermore, elevated TG, which contain three acyl chains, demonstrated a total acyl-chain length surpassing 60 carbon atoms, with up to eight double bonds. Our results also revealed a relationship between lipid fold increase and total acyl-chain length. In ALD individuals, an increase in total acyl-chain length was associated with a larger fold increase (Fig. 3A).

After identifying trends in the total acyl-chain length of lipids that were elevated in ALD, we sought to determine the specific variants of VLCFA present in these lipids by using the acquired MS/MS data. Our data unveiled an array of VLCFA with different chain lengths, predominantly containing either zero or one double bond, that are integrated into various lipid classes in ALD (Fig. 3C). LPC, PC, and TG were the lipid classes that presented the highest number of detected VLCFA-containing species (Fig. 3C). Notably, C26:1 was the most frequently detected VLCFA, followed by C26:0. Our analysis identified 31 VLCFA containing species in the LPC class, with a chain length of up to 29 carbon atoms. Our method is capable of chromatographically separating SN1 and SN2 isomers of LPC lipids. For 6 VLCFA, both an SN1 or SN2 LPC variant was detected including LPC 22:3, LPC 22:6, LPC 24:0, LPC 24:2, LPC 26:0 and LPC 26:1 (Supplemental Table S1). It is worth noting that distinguishing between these variants based solely on ion mobility and poses a notable challenge, given the similarity in their CCS values. In the PC class, 37 species were discovered with VLCFA, having up to 30 total carbon atoms. Similarly, we identified 123 TG species containing VLCFA. Detected polyunsaturated VLCFA were predominantly 22 carbon atoms in length, while VLCFA longer than 22 carbon atoms were primarily saturated and monounsaturated VLCFA. Interestingly, certain TG lipids encapsulated more than one VLCFA within a single lipid molecule, such as TG 18:1_26:0_26:0 and TG 16:0_26:0_26:1. In lipid molecules with multiple acyl chains, VLCFA were mostly combined with C16:0, C16:1, C18:0, and C18:1 acyl chains.

To assess the abundance of different VLCFA within a specific lipid class, we aggregated the relative abundances of lipid species containing specific VLCFA within the LPC, PC, and TG lipid classes. For example, in Fig. 3D, C26:0 in PC represents the combined abundance of all PC lipids containing C26:0. C26:1, followed by C26:0 and C24:1, was the most abundantly detected VLCFA across LPC, PC, and TG in ALD samples. VLCFA that were 22 carbon atoms in length did not show any discernible difference in abundance between ALD and control samples. However, for VLCFA with chain lengths of 24 carbon atoms and beyond, a distinct difference in abundance was observed between ALD and controls. This difference was most evident for VLCFA possessing zero or one double bond. Interestingly, the disparity in VLCFA abundance between ALD and control samples increased with chain length. Furthermore, as the chain length increased, the abundance of these VLCFA decreased (Fig. 3D). Collectively, these results show that elongation products of saturated and monounsaturated VLCFA ranging from C24-C30 are incorporated into complex lipids, primarily in LPC, PC, and TG.

### 4D-lipidomics allows for the identification of new biomarker candidates in ALD

**Fig. 4:**
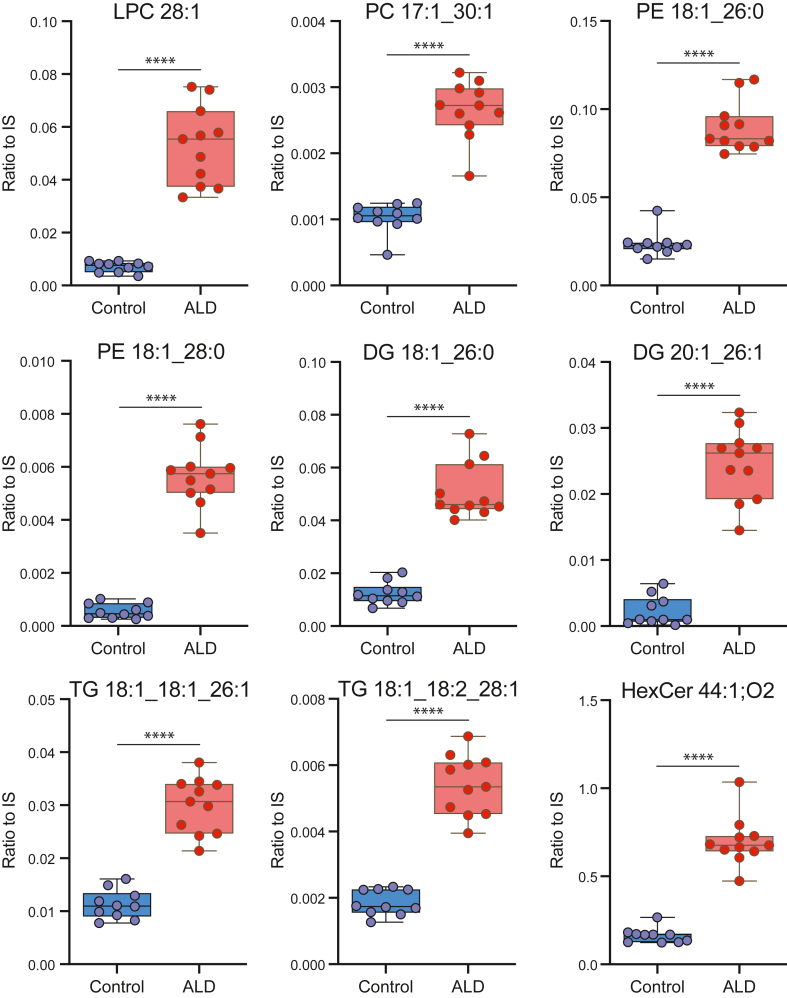
4D-lipidomics allows for the identification of new biomarker candidates in ALD. Examples of biomarker candidates in ALD are presented. Lipid abundances were calculated by dividing the analyte response by that of the corresponding internal standard (ratio to IS). (∗∗∗∗*P* < 0.0001). ALD, adrenoleukodystrophy.

LPC 26:0 stands as a well-established marker in newborn screening and proves invaluable in diagnosing ALD individuals. Nonetheless, there is currently no biomarker for the identification of high-risk individuals for cerebral ALD before symptoms arise, impeding personalized follow-up. Furthermore, the unavailability of biomarkers for disease monitoring, correlating with disease severity and tracking progression, remains a limitation. There is therefore a pressing need to identify additional biomarkers that can complement LPC 26:0 in the diagnosis, characterization, and prognostication of ALD. To facilitate the identification of new biomarker candidates, we examined our data for lipids that not only exhibited a significant deviation in ALD when compared to controls, but also manifested nonoverlapping values. This investigation yielded 121 candidate biomarker exhibiting elevated levels in ALD (Supplemental Table S3). As expected, these candidates included species containing VLCFA and spanned lipid classes such as PC, TG, DG, CE, and HexCer. While a variety of lipid species demonstrated lower levels in ALD, none of these species had nonoverlapping values with controls, making them unsuited as ALD biomarkers. Figure 4 provides a visual representation of nine biomarker candidates from the PC, TG, DG, and HexCer classes. These biomarkers warrant further investigation to ascertain their potential in offering valuable insights into the complex and diverse nature of ALD.

## Discussion

ALD is a monogenic disorder that exhibits a wide spectrum of clinical features and is characterized by the accumulation of VLCFA in body fluids and tissues. The study of VLCFA accumulation in ALD has predominantly centered around the analysis of C22:0, C24:0, and C26:0 fatty acids using GC-MS. However, this analysis does not shed light on the biomolecular origins of VLCFA. Moreover, it lacks the capability to assess the overall impact of VLCFA accumulation on the full lipid profile. This study aimed to characterize impact of VLCFA accumulation on the ALD lipidome using 4D-lipidomics. To accomplish this, we compared the lipidome of fibroblasts derived from ALD individuals with those from healthy controls. Our 4D-lipidomics method uses PASEF scan mode which synchronizes ion mobility separation with MS/MS precursor selection. This allowed for high-speed MS/MS acquisition rates and ion mobility resolved MS/MS spectra. Using this approach, we were able to detect 1,155 lipid species across a broad range of classes including LPC, PC, TG, SM, CE, and others. Furthermore, lipid annotation certainty was enhanced by the evaluation using four distinct dimensions: RT, CCS, m/z, and MS/MS spectra. While 4D-lipidomics does not inherently yield data that are unattainable by other methods, it offers an effective means to characterize the lipidome of ALD fibroblasts.

Our findings demonstrate a significant increase in multiple lipids within the ALD lipidome compared to healthy controls. LPC 26:0, the marker used in ALD newborn screening was one of the most prominently elevated lipids. Additionally, numerous other LPC species and species from different lipid classes, such as PC, TG, SM, and CE, were found to be elevated in ALD fibroblasts. These observations demonstrate that the alterations seen in ALD go beyond a single lipid class and likely impacts a wide range of lipid-mediated physiological processes.

By analyzing the total chain length of the elevated lipids in ALD, we found that these lipids likely incorporated VLCFA. Moreover, the fold-change of these lipids in ALD samples compared to control samples increased with longer total acyl-chain lengths. Our findings demonstrate that an array of VLCFA, varying in length, are incorporated into lipids in ALD-affected individuals. We found that the highest number of VLCFA species was detected in LPC, PC, and TG. In these lipid classes, species containing C26:1, C26:0 were the most frequently detected ones with the highest abundancies. The abundance of VLCFA is influenced by a variety of metabolic processes and is directly impacted by the activity of the enzymes integral to these pathways (32). The accumulation of VLCFA in ALD is not solely due to dietary intake. Tsuji *et al.* demonstrated that the majority of VLCFA in ALD individuals is synthesized endogenously (33). ELOVL1 (elongation of very long chain fatty acids protein 1) primarily regulates the synthesis of VLCFA by elongating saturated (C22:0 – C26:0) and monounsaturated (C22:1 – C26:1) acyl-CoA fatty acids. Notably, in ALD individuals, there is an increase in the elongation of both saturated and monounsaturated VLCFA. (34) This aligns with our findings, as we detected significant elevated levels of saturated and monounsaturated VLCFA-containing lipid species in ALD. Interestingly, pharmacological inhibition of ELOVL1 has been identified as an effective mechanism to reduce the production and resulting concentration of C26:0 (32, 35). VLCFA levels are also influenced by the conversion of VLCFA into dicarboxylic-VLCFA through the process of ω-oxidation, facilitated by the enzymes CYP4F2 and CYP4F3B. (36, 37) This could serve as a possible escape route for VLCFA as dicarboxylic-VLCFA is transported into peroxisomes via ABCD3. Consequently, the β-oxidation of dicarboxylic-VLCFA is normal in ALD individuals. (38) Changes in the functionality of key enzymes involved in VLCFA metabolism can directly influence VLCFA abundance. For example, the genetic variant p.V433M of CYP4F2 results in reduced enzyme levels, leading to a decreased conversion of VLCFA into VLCFA-dicarboxylic acids through ω-oxidation. (39) In this study, we identified VLCFA with varying chain lengths and degrees of saturation. VLCFA with chain lengths exceeding 22 carbon atoms displayed zero or one double bond. The chain length and degree of saturation of VLCFA are critical in dictating the effect of their accumulation. For example, research on ALD fibroblasts demonstrated that saturated VLCFA induce endoplasmic reticulum stress, unlike their unsaturated counterparts. (40) The enzyme stearoyl-CoA desaturase-1 (SCD1), which regulates the saturation level of long-chain fatty acids, significantly influences VLCFA concentrations. (41) Pharmacological activation of SCD1 reduces the synthesis of saturated VLCFA, whereas inhibiting SCD1 increases saturated VLCFA levels. (41) The impact of specific genetic variants and the regulatory mechanisms of enzymes crucial to VLCFA metabolism on VLCFA-containing lipids is currently unexplored territory, offering an intriguing avenue for future research.

In this study, PC and LPC were two of the most affected lipid classes in ALD. We found that PC contained VLCFA up to 30 carbon atoms. In these lipids, VLCFA were typically combined with acyl chains containing 16 or 18 carbon atoms. This could reflect a compensation mechanism where the effect of VLCFA on the total length of the lipid is minimized by combining it with a shorter acyl chain. PC is the major component of cell membranes, playing a crucial role in preserving their structure and functionality. The incorporation of VLCFA into PC lipids likely has a significant impact on membrane structure, fluidity, permeability, protein-lipid interactions, and cellular signaling, collectively influencing cellular functions. VLCFA-containing PC lipids have been associated with the development of cerebral lesions in ALD. An analysis of postmortem brains from cerebral ALD cases by Theda *et al.* revealed a significant enrichment of VLCFA in the PC fraction, both in the active demyelinating area and in intact white matter (20). CEs containing VLCFA were found only in the active demyelinating area. This indicates that the increase in VLCFA levels within PC precedes the onset of cerebral demyelination. The presence of VLCFA-containing PC in the myelin membrane could potentially alter its structural integrity, leading to immunologically mediated destruction of myelin. LPC, a degradation product of PC, can be generated intracellularly by the actions of phospholipase A_1_ (PLA1) and phospholipase A_2_ (PLA2). (42) In the presence of acyl-CoA, LPC can be reconverted to PC by the enzyme lysophosphatidylcholine acyltransferase in a process known as the Lands cycle. Extracellularly, LPC can be produced by the actions of secreted phospholipase A_2_ or lecithin–cholesterol acyltransferase as they transfer fatty acids to free cholesterol. Notably, LPC is increasingly linked with cardiovascular and neurodegenerative diseases, including Alzheimer's disease. (43) Furthermore, LPC enriched with VLCFA has been found to be cytotoxic. (44) The intracortical injection of LPC 24:0 induced extensive microglial activation and apoptosis in WT mice. (44).

Multiple VLCFA variants were also detected in neutral lipids including CE, DG, and TG. Interestingly, for TG we observed multiple VLCFA in a single lipid. TG plays crucial roles in energy storage and metabolism. Incorporation of these fatty acids into TG lipids could have significant implications for their function, potentially disrupting cellular lipid metabolism, lipid droplet formation, and degradation. The incorporation of VLCFA in TG and CE might be reflective of a protective (or deflective) mechanism against fatty acid-induced lipotoxicity by storing toxic fatty acids in lipid droplets. (45) Moreover, VLCFAs-containing CE have been linked to ALD-related adrenal insufficiency as VLCFA buildup in CE is associated with adrenal cortex apoptosis and atrophy, disrupting cortisol production (46).

The current inability to establish a direct link between genotype and phenotype in individuals with ALD poses a significant challenge in predicting clinical outcomes. Additionally, the absence of reliable molecular markers for assessing disease severity hinders accurate prognoses. (47) Consequently, there is an urgent need for sensitive biomarkers that can effectively monitor disease progression and evaluate the effectiveness of therapeutic interventions. To address this need, our 4D-lipidomics approach has identified 121 promising new biomarker candidates for ALD. Notably, these biomarkers all contain VLCFA and consisted mostly of LPC, PC, and TG lipids. This aligns closely with earlier lipidomic studies focused on ALD. Raas *et al.* and Herzog *et al.*, through their analysis on fibroblasts, reported elevated levels of VLCFA containing LPC lipids and PC lipids in ALD compared to healthy controls (41, 48). Furthermore, Huffnagel *et al.* performed lipidomic analysis of plasma samples from 20 women diagnosed with ALD. (49) Interestingly, the results revealed marked elevations in similar LPC and PC lipids in ALD. In addition, Richmond *et al.*, in their study of plasma from six pairs of brothers affected by ALD, also observed increases in LPC and PC lipids, further substantiating the consistent lipidomic alterations associated with the ALD. (50) Leveraging the availability of extensive biological sample repositories and the implementation of newborn screening programs (51, 52, 53), there is a unique opportunity to evaluate the potential of these biomarker candidates. While our study identified these candidates within fibroblast samples, it is imperative to subject them to validation across a range of sample matrices, including plasma and cerebrospinal fluid. Expanding the evaluation of these biomarker candidates beyond fibroblasts and into bodily fluids such as plasma and cerebrospinal fluid will fortify their validity as indicators suitable for a broader clinical context, enhancing their potential as reliable tools for the detection and monitoring of ALD. Our ongoing work focuses on validating these biomarkers across diverse sample matrices, including plasma and blood spots, to establish their effectiveness in a wider clinical context.

## Conclusion

In conclusion, our study used a comprehensive 4D-lipidomics approach to thoroughly characterize the lipidome of ALD fibroblasts. Through our analysis, we demonstrated the incorporation of a range of VLCFA into various lipid classes and identified new biomarker candidates in fibroblasts. Collectively, this investigation highlights the power of 4D-lipidomics in uncovering novel insights into the intricate lipid alterations occurring in this metabolic disorder. The transfer of this 4D-lipidomics approach to other disease contexts using different sample types such as tissue or plasma is subject of our ongoing research.

## Data Availability

All data used in the article are available from the corresponding author upon reasonable request.

## Supplemental data

This article contains supplemental data.

## Conflict of interest

Sven W. Meyer is employed at Bruker Daltonics GmbH. All the other authors declarethat they have no conflict of interest with the contents of this article.
