# Supplementary material for: Four-dimensional lipidomics profiling in X-linked adrenoleukodystrophy using trapped ion mobility mass spectrometry
Source: J Lipid Res. 2024 May 23;65(6):100567. doi: 10.1016/j.jlr.2024.100567 (PMC11234049; doi:10.1016/j.jlr.2024.100567)
Supplement: Supplemental Data [file mmc3.pdf]

Separation Workflow

Overall study design

Title of the study	Four-dimensional Lipidomics Profiling in X-linked Adrenoleukodystrophy using Trapped Ion Mobility Mass Spectrometry		
Document creation date	04/15/2024	Corresponding Email	y.r.jaspers@amsterdamumc.nl
Principle investigator	Yorrick R.J. Jaspers	Is the workflow targeted or untargeted?	Untargeted
Institution	Amsterdam UMC	Clinical	No

Lipid extraction

Extraction method	1-phase system	1-phase system	1:1 (v/v) methanol:chloroform
Derivatization	-	Were internal standards added prior extraction?	Yes
pH adjustment	None		

Analytical platform

Ionizaton additives	Ammonium acetate, Formic acid	MS Level	MS2
Number of separation dimensions	One dimension	Mass window for precursor ion isolation (in Da total isolation window)	1
Separation type 1	LC	Mass resolution for detected ion at MS2	High resolution
Separation mode 1 (liquid)	RP	Resolution at m/z 200 at MS2	60000
Detector	Mass spectrometer	Mass accuracy in ppm at MS2	4
MS type	timsTOF Pro2	Recording mode of raw data at MS2	Profile mode
MS vendor	Bruker	Was/Were additional dimension/techniques used	Yes
Ion source	HESI		

Quality control

Blanks	Yes	Quality control	Yes
Type of Blanks	Extraction blank, Solvent blank	Type of QC sample	Sample pool

Method qualification and validation

Method validation	No

Reporting

Are reported raw data uploaded into repository?	Available on request	Raw data upload	Available on request
Are metadata available?	Available on request	Additional comments	-

Sample Descriptions

Fibroblasts / Human / Cells

Provided information	-	Additives	None
Temperature handling original sample	Unknown	Were samples stored under inert gas?	No
Instant sample preparation	No	Additional preservation methods	No
Storage temperature	-80 °C	Biobank samples	No

Lipid Class Descriptions

1) LPC[M+H]+ / Lipid identification

Lipid class	LPC	Limit of detection	No
MS Level for identification	MS1, MS2	RT verified by standard	Yes
Identification level	sn Position	Separation of isobaric/isomeric interferece confirmed	No
Polarity mode	Positive	Model for separation prediction	No
Type of positive (precursor)ion	[M+H]+	Additional dimension/techniques	IMS
Fragments for identification	Fragment name: HG(PC,184)	CCS verified by standard	No
Isotope correction at MS1	No	How was/were the additional dimension(s) used?	CCS was used (in combination with other parameters) in the identification of lipids, separation of potential isobaric/isomeric interference.
Isotope correction at MS2	No	Was a model used to predict lipid molecule separation?	No
MS1 verified by standard	Yes	Lipid Identification Software	Lipid identification was done using MetaboScape 2023b (Bruker Daltonics) utilizing an in-house generated retention time and m/z database, rule-based lipid annotation and the MS DIAL MS/MS library LipidBlast (version 68).
MS2 verified by standard	Yes	Data manipulation	-
Background check at MS1	Yes	Nomenclature for intact lipid molecule	Yes
Background check at MS2	No	Nomenclature for fragment ions	N/A
Did you presume assumptions for identification?	No	Further identification remarks	For lipid identification, the Lipidomics Standards Initiative guidelines were followed, employing lipid class-specific fragments to determine the lipid species and molecular lipid species-specific fragments for the annotation of hydrocarbon chains
Check isomer overlap	Yes		

1) LPC[M+H]+ / Lipid quantification

Quantitative	No	Batch correction	No
Normalization to reference	No	Further quantification remarks	The reported lipid abundances were semi-quantitative, calculated by dividing the analyte response by that of the corresponding internal standard.

2) PC[M+H]+ / Lipid identification

Lipid class	PC	Limit of detection	No
MS Level for identification	MS1, MS2	RT verified by standard	Yes
Identification level	Molecular species level	Separation of isobaric/isomeric interferece confirmed	No
Polarity mode	Positive	Model for separation prediction	No
Type of positive (precursor)ion	[M+H]+	Additional dimension/techniques	IMS
Fragments for identification		CCS verified by standard	No

Fragment name
HG(PC,184)
-FA1 (+HO)
-FA1 (-H)
-FA2 (+HO)
-FA2 (-H)

Isotope correction at MS1	No	How was/were the additional dimension(s) used?	CCS was used (in combination with other parameters) in the identification of lipids, separation of potential isobaric/isomeric interference.
Isotope correction at MS2	No	Was a model used to predict lipid molecule separation?	No
MS1 verified by standard	Yes	Lipid Identification Software	Lipid identification was done using MetaboScape 2023b (Bruker Daltonics) utilizing an in-house generated retention time and m/z database, rule-based lipid annotation and the MS DIAL MS/MS library LipidBlast (version 68).
MS2 verified by standard	No	Data manipulation	-
Background check at MS1	Yes	Nomenclature for intact lipid molecule	Yes
Background check at MS2	No	Nomenclature for fragment ions	N/A
Did you presume assumptions for identification?	No	Further identification remarks	For lipid identification, the Lipidomics Standards Initiative guidelines were followed, employing lipid class-specific fragments to determine the lipid species and molecular lipid species-specific fragments for the annotation of hydrocarbon chains.
Check isomer overlap	Yes		

2) PC[M+H]+ / Lipid quantification

Quantitative	No	Batch correction	No
Normalization to reference	No	Further quantification remarks	The reported lipid abundances were semi-quantitative, calculated by dividing the analyte response by that of the corresponding internal standard.

3) TG[M+NH4]+ / Lipid identification

Lipid class	TG	Limit of detection	No
MS Level for identification	MS1, MS2	RT verified by standard	Yes
Identification level	Molecular species level	Separation of isobaric/isomeric interferece confirmed	No
Polarity mode	Positive	Model for separation prediction	No
Type of positive (precursor)ion	[M+NH4]+	Additional dimension/techniques	IMS
Fragments for identification	Fragment name: -FA(+HO) -TG(17); -TG(17)	CCS verified by standard	No
Isotope correction at MS1	No	How was/were the additional dimension(s) used?	CCS was used (in combination with other parameters) in the identification of lipids, separation of potential isobaric/isomeric interference.
Isotope correction at MS2	No	Was a model used to predict lipid molecule separation?	No
MS1 verified by standard	Yes	Lipid Identification Software	Lipid identification was done using MetaboScape 2023b (Bruker Daltonics) utilizing an in-house generated retention time and m/z database, rule-based lipid annotation and the MS DIAL MS/MS library LipidBlast (version 68).
MS2 verified by standard	No	Data manipulation	-
Background check at MS1	Yes	Nomenclature for intact lipid molecule	Yes
Background check at MS2	No	Nomenclature for fragment ions	N/A
Did you presume assumptions for identification?	No	Further identification remarks	For lipid identification, the Lipidomics Standards Initiative guidelines were followed, employing lipid class-specific fragments to determine the lipid species and molecular lipid species-specific fragments for the annotation of hydrocarbon chains.
Check isomer overlap	Yes		

3) TG[M+NH4]+ / Lipid quantification

Quantitative	No	Batch correction	No
Normalization to reference	No	Further quantification remarks	The reported lipid abundances were semi-quantitative, calculated by dividing the analyte response by that of the corresponding internal standard.

4) DG[M+NH4]+ / Lipid identification

Lipid class	DG	Limit of detection	No
MS Level for identification	MS1, MS2	RT verified by standard	Yes
Identification level	Molecular species level	Separation of isobaric/isomeric interferece confirmed	No
Polarity mode	Positive	Model for separation prediction	Yes
Type of positive (precursor)ion	[M+NH4]+	Additional dimension/techniques	IMS
Fragments for identification	Fragment name: -(H2O+NH3,35); -FA1(-H)-(H2O+NH3); -FA2(-H)-(H2O+NH3)	CCS verified by standard	No
Isotope correction at MS1	No	How was/were the additional dimension(s) used?	CCS was used (in combination with other parameters) in the identification of lipids, separation of potential isobaric/isomeric interference.
Isotope correction at MS2	No	Was a model used to predict lipid molecule separation?	No
MS1 verified by standard	Yes	Lipid Identification Software	Lipid identification was done using MetaboScape 2023b (Bruker Daltonics) utilising an in-house generated retention time and m/z database, rule-based lipid annotation and the MS DIAL MS/MS library LipidBlast (version 68).
MS2 verified by standard	No	Data manipulation	-
Background check at MS1	Yes	Nomenclature for intact lipid molecule	Yes
Background check at MS2	No	Nomenclature for fragment ions	N/A
Did you presume assumptions for identification?	No	Further identification remarks	For lipid identification, the Lipidomics Standards Initiative guidelines were followed, employing lipid class-specific fragments to determine the lipid species and molecular lipid species-specific fragments for the annotation of hydrocarbon chains.
Check isomer overlap	Yes		

4) DG[M+NH4]+ / Lipid quantification

Quantitative	No	Batch correction	No
Normalization to reference	No	Further quantification remarks	The reported lipid abundances were semi-quantitative, calculated by dividing the analyte response by that of the corresponding internal standard.

5) CE[M+NH4]+ / Lipid identification

Lipid class	CE	Limit of detection	No
MS Level for identification	MS1, MS2	RT verified by standard	Yes
Identification level	Molecular species level	Separation of isobaric/isomeric interferece confirmed	No
Polarity mode	Positive	Model for separation prediction	No
Type of positive (precursor)ion	[M+NH4]+	Additional dimension/techniques	IMS
Fragments for identification<br>**Fragment name**<br>-FA1 (+HO) -CE(17)		CCS verified by standard	No
Isotope correction at MS1	No	How was/were the additional dimension(s) used?	CCS was used (in combination with other parameters) in the identification of lipids, separation of potential isobaric/isomeric interference.
Isotope correction at MS2	No	Was a model used to predict lipid molecule separation?	No
MS1 verified by standard	Yes	Lipid Identification Software	Lipid identification was done using MetaboScape 2023b (Bruker Daltonics) utilizing an in-house generated retention time and m/z database, rule-based lipid annotation and the MS DIAL MS/MS library LipidBlast (version 68).
MS2 verified by standard	Yes	Data manipulation	-
Background check at MS1	Yes	Nomenclature for intact lipid molecule	Yes
Background check at MS2	No	Nomenclature for fragment ions	N/A
Did you presume assumptions for identification?	No	Further identification remarks	For lipid identification, the Lipidomics Standards Initiative guidelines were followed, employing lipid class-specific fragments to determine the lipid species and molecular lipid species-specific fragments for the annotation of hydrocarbon chains.
Check isomer overlap	Yes		

5) CE[M+NH4]+ / Lipid quantification

Quantitative	No	Batch correction	No
Normalization to reference	No	Further quantification remarks	The reported lipid abundances were semi-quantitative, calculated by dividing the analyte response by that of the corresponding internal standard.

6) Cer[M+H]+ / Lipid identification

Lipid class	Cer	Limit of detection	No
MS Level for identification	MS1, MS2	RT verified by standard	Yes
Identification level	Molecular species level	Separation of isobaric/isomeric interferece confirmed	No
Polarity mode	Positive	Model for separation prediction	No
Type of positive (precursor)ion	[M+H]+	Additional dimension/techniques	IMS
Fragments for identification		CCS verified by standard	No

Fragment name
-(H2O,18)
LCB(-HO)
LCB(-H3O2)
LCB(-CH3O2)

Isotope correction at MS1	No	How was/were the additional dimension(s) used?	CCS was used (in combination with other parameters) in the identification of lipids, separation of potential isobaric/isomeric interference.
Isotope correction at MS2	No	Was a model used to predict lipid molecule separation?	No
MS1 verified by standard	Yes	Lipid Identification Software	Lipid identification was done using MetaboScape 2023b (Bruker Daltonics) utilizing an in-house generated retention time and m/z database, rule-based lipid annotation and the MS DIAL MS/MS library LipidBlast (version 68).
MS2 verified by standard	No	Data manipulation	-
Background check at MS1	Yes	Nomenclature for intact lipid molecule	Yes
Background check at MS2	No	Nomenclature for fragment ions	N/A
Did you presume assumptions for identification?	No	Further identification remarks	For lipid identification, the Lipidomics Standards Initiative guidelines were followed, employing lipid class-specific fragments to determine the lipid species and molecular lipid species-specific fragments for the annotation of hydrocarbon chains.
Check isomer overlap	Yes		

6) Cer[M+H]+ / Lipid quantification

Quantitative	No	Batch correction	No
Normalization to reference	No	Further quantification remarks	The reported lipid abundances were semi-quantitative, calculated by dividing the analyte response by that of the corresponding internal standard.

7) AC[M+H]+ / Lipid identification

Lipid class	AC	RT verified by standard	No
MS Level for identification	MS1	Separation of isobaric/isomeric interferece confirmed	No
Identification level	Species level	Model for separation prediction	No
Polarity mode	Positive	Additional dimension/techniques	IMS
Type of positive (precursor)ion	[M+H]+	CCS verified by standard	No
Isotope correction at MS1	No	How was/were the additional dimension(s) used?	CCS was used (in combination with other parameters) in the identification of lipids, separation of potential isobaric/isomeric interference.
MS1 verified by standard	No	Was a model used to predict lipid molecule separation?	No
Background check at MS1	Yes	Lipid Identification Software	Lipid identification was done using MetaboScape 2023b (Bruker Daltonics) utilizing an in-house generated retention time and m/z database, rule-based lipid annotation and the MS DIAL MS/MS library LipidBlast (version 68).
Did you presume assumptions for identification?	No	Data manipulation	-
Check isomer overlap	Yes	Nomenclature for intact lipid molecule	Yes
Limit of detection	No	Further identification remarks	For lipid identification, the Lipidomics Standards Initiative guidelines were followed, employing lipid class-specific fragments to determine the lipid species and molecular lipid species-specific fragments for the annotation of hydrocarbon chains.

7) AC[M+H]+ / Lipid quantification

Quantitative	No	Batch correction	No
Normalization to reference	No	Further quantification remarks	The reported lipid abundances were semi-quantitative, calculated by dividing the analyte response by that of the corresponding internal standard.

8) DG O[M+NH4]+ / Lipid identification

Lipid class	DG O	Limit of detection	No
MS Level for identification	MS1, MS2	RT verified by standard	No
Identification level	Molecular species level	Separation of isobaric/isomeric interferece confirmed	Yes
Polarity mode	Positive	Model for separation prediction	No
Type of positive (precursor)ion	[M+NH4]+	Additional dimension/techniques	IMS
Fragments for identification	Fragment name: -FA (-H)-(H2O+NH3); -(H2O+NH3,35)	CCS verified by standard	No
Isotope correction at MS1	No	How was/were the additional dimension(s) used?	CCS was used (in combination with other parameters) in the identification of lipids, separation of potential isobaric/isomeric interference.
Isotope correction at MS2	No	Was a model used to predict lipid molecule separation?	No
MS1 verified by standard	No	Lipid Identification Software	Lipid identification was done using MetaboScape 2023b (Bruker Daltonics) utilizing an in-house generated retention time and m/z database, rule-based lipid annotation and the MS DIAL MS/MS library LipidBlast (version 68).
MS2 verified by standard	No	Data manipulation	-
Background check at MS1	Yes	Nomenclature for intact lipid molecule	Yes
Background check at MS2	No	Nomenclature for fragment ions	N/A
Did you presume assumptions for identification?	No	Further identification remarks	For lipid identification, the Lipidomics Standards Initiative guidelines were followed, employing lipid class-specific fragments to determine the lipid species and molecular lipid species-specific fragments for the annotation of hydrocarbon chains.
Check isomer overlap	Yes		

8) DG O[M+NH4]+ / Lipid quantification

Quantitative	No	Batch correction	No
Normalization to reference	No	Further quantification remarks	-

9) Hex2Cer[M+H]+ / Lipid identification

Lipid class	Hex2Cer	Limit of detection	No
MS Level for identification	MS1, MS2	RT verified by standard	Yes
Identification level	Species level	Separation of isobaric/isomeric interferece confirmed	No
Polarity mode	Positive	Model for separation prediction	No
Type of positive (precursor)ion	[M+H]+	Additional dimension/techniques	IMS
Fragments for identification		CCS verified by standard	No

Fragment name
-(H2O,18)
LCB(-HO)
LCB(-H3O2)
LCB(-CH3O2)
-HG(Hex2,342)
-HG(Hex2,360)
-HG(Hex2,324)

Isotope correction at MS1	No	How was/were the additional dimension(s) used?	CCS was used (in combination with other parameters) in the identification of lipids, separation of potential isobaric/isomeric interference.
Isotope correction at MS2	No	Was a model used to predict lipid molecule separation?	No
MS1 verified by standard	No	Lipid Identification Software	Lipid identification was done using MetaboScape 2023b (Bruker Daltonics) utilizing an in-house generated retention time and m/z database, rule-based lipid annotation and the MS DIAL MS/MS library LipidBlast (version 68).
MS2 verified by standard	No	Data manipulation	-
Background check at MS1	Yes	Nomenclature for intact lipid molecule	Yes
Background check at MS2	No	Nomenclature for fragment ions	N/A
Did you presume assumptions for identification?	No	Further identification remarks	For lipid identification, the Lipidomics Standards Initiative guidelines were followed, employing lipid class-specific fragments to determine the lipid species and molecular lipid species-specific fragments for the annotation of hydrocarbon chains.
Check isomer overlap	Yes		

9) Hex2Cer[M+H]+ / Lipid quantification

Quantitative	No	Batch correction	No
Normalization to reference	No	Further quantification remarks	The reported lipid abundances were semi-quantitative, calculated by dividing the analyte response by that of the corresponding internal standard.

10) Hex3Cer[M+H]+ / Lipid identification

Lipid class	Hex3Cer	Limit of detection	No
MS Level for identification	MS1, MS2	RT verified by standard	No
Identification level	Species level	Separation of isobaric/isomeric interferece confirmed	No
Polarity mode	Positive	Model for separation prediction	No
Type of positive (precursor)ion	[M+H]+	Additional dimension/techniques	IMS
Fragments for identification		CCS verified by standard	No

Fragment name
-(H2O,18)
LCB(-HO)
LCB(-H3O2)
LCB(-CH3O2)
-HG(Hex3,504)
-HG(Hex3,522)
-HG(Hex3,534)
-HG(Hex2,342)
-HG(Hex,180)

Isotope correction at MS1	No	How was/were the additional dimension(s) used?	CCS was used (in combination with other parameters) in the identification of lipids, separation of potential isobaric/isomeric interference.
Isotope correction at MS2	No	Was a model used to predict lipid molecule separation?	No
MS1 verified by standard	No	Lipid Identification Software	Lipid identification was done using MetaboScape 2023b (Bruker Daltonics) utilizing an in-house generated retention time and m/z database, rule-based lipid annotation and the MS DIAL MS/MS library LipidBlast (version 68).
MS2 verified by standard	No	Data manipulation	-
Background check at MS1	Yes	Nomenclature for intact lipid molecule	Yes
Background check at MS2	No	Nomenclature for fragment ions	N/A
Did you presume assumptions for identification?	No	Further identification remarks	For lipid identification, the Lipidomics Standards Initiative guidelines were followed, employing lipid class-specific fragments to determine the lipid species and molecular lipid species-specific fragments for the annotation of hydrocarbon chains.
Check isomer overlap	Yes		

10) Hex3Cer[M+H]+ / Lipid quantification

Quantitative	No	Batch correction	No
Normalization to reference	No	Further quantification remarks	The reported lipid abundances were semi-quantitative, calculated by dividing the analyte response by that of the corresponding internal standard.

11) HexCer[M+H]+ / Lipid identification

Lipid class	HexCer	Limit of detection	No
MS Level for identification	MS1, MS2	RT verified by standard	Yes
Identification level	Species level	Separation of isobaric/isomeric interferece confirmed	No
Polarity mode	Positive	Model for separation prediction	No
Type of positive (precursor)ion	[M+H]+	Additional dimension/techniques	IMS
Fragments for identification		CCS verified by standard	No

Fragment name
-(H2O,18)
LCB(-HO)
LCB(-H3O2)
LCB(-CH3O2)
-HG(Hex,180)
-HG(Hex,198)
-HG(Hex,162)

Isotope correction at MS1	No	How was/were the additional dimension(s) used?	CCS was used (in combination with other parameters) in the identification of lipids, separation of potential isobaric/isomeric interference.
Isotope correction at MS2	No	Was a model used to predict lipid molecule separation?	No
MS1 verified by standard	Yes	Lipid Identification Software	Lipid identification was done using MetaboScape 2023b (Bruker Daltonics) utilising an in-house generated retention time and m/z database, rule-based lipid annotation and the MS DIAL MS/MS library LipidBlast (version 68).
MS2 verified by standard	No	Data manipulation	-
Background check at MS1	Yes	Nomenclature for intact lipid molecule	Yes
Background check at MS2	No	Nomenclature for fragment ions	N/A
Did you presume assumptions for identification?	No	Further identification remarks	For lipid identification, the Lipidomics Standards Initiative guidelines were followed, employing lipid class-specific fragments to determine the lipid species and molecular lipid species-specific fragments for the annotation of hydrocarbon chains.
Check isomer overlap	Yes		

11) HexCer[M+H]+ / Lipid quantification

Quantitative	No	Batch correction	No
Normalization to reference	No	Further quantification remarks	The reported lipid abundances were semi-quantitative, calculated by dividing the analyte response by that of the corresponding internal standard.

12) LPE[M-H]- / Lipid identification

Lipid class	LPE	Limit of detection	No
MS Level for identification	MS1, MS2	RT verified by standard	Yes
Identification level	Species level	Separation of isobaric/isomeric interferece confirmed	No
Polarity mode	Negative	Model for separation prediction	No
Type of negative (precursor)ion	[M-H]-	Additional dimension/techniques	IMS
Fragments for identification		CCS verified by standard	No

Fragment name
-FA1(-H)-(H2O)
-FA1(-H)
GP(153)
FA1(+O)

Isotope correction at MS1	No	How was/were the additional dimension(s) used?	CCS was used (in combination with other parameters) in the identification of lipids, separation of potential isobaric/isomeric interference.
Isotope correction at MS2	No	Was a model used to predict lipid molecule separation?	No
MS1 verified by standard	Yes	Lipid Identification Software	Lipid identification was done using MetaboScape 2023b (Bruker Daltonics) utilising an in-house generated retention time and m/z database, rule-based lipid annotation and the MS DIAL MS/MS library LipidBlast (version 68).
MS2 verified by standard	No	Data manipulation	-
Background check at MS1	Yes	Nomenclature for intact lipid molecule	Yes
Background check at MS2	No	Nomenclature for fragment ions	N/A
Did you presume assumptions for identification?	No	Further identification remarks	For lipid identification, the Lipidomics Standards Initiative guidelines were followed, employing lipid class-specific fragments to determine the lipid species and molecular lipid species-specific fragments for the annotation of hydrocarbon chains.
Check isomer overlap	Yes		

12) LPE[M-H]- / Lipid quantification

Quantitative	No	Batch correction	No
Normalization to reference	No	Further quantification remarks	The reported lipid abundances were semi-quantitative, calculated by dividing the analyte response by that of the corresponding internal standard.

13) LPE[M+H]+ / Lipid identification

Lipid class	LPE	Limit of detection	No
MS Level for identification	MS1, MS2	RT verified by standard	Yes
Identification level	Species level	Separation of isobaric/isomeric interferece confirmed	No
Polarity mode	Positive	Model for separation prediction	No
Type of positive (precursor)ion	[M+H]+	Additional dimension/techniques	IMS
Fragments for identification<br>**Fragment name**<br>-HG(PE,141)		CCS verified by standard	No
Isotope correction at MS1	No	How was/were the additional dimension(s) used?	CCS was used (in combination with other parameters) in the identification of lipids, separation of potential isobaric/isomeric interference.
Isotope correction at MS2	No	Was a model used to predict lipid molecule separation?	No
MS1 verified by standard	Yes	Lipid Identification Software	Lipid identification was done using MetaboScape 2023b (Bruker Daltonics) utilising an in-house generated retention time and m/z database, rule-based lipid annotation and the MS DIAL MS/MS library LipidBlast (version 68).
MS2 verified by standard	No	Data manipulation	-
Background check at MS1	Yes	Nomenclature for intact lipid molecule	Yes
Background check at MS2	No	Nomenclature for fragment ions	N/A
Did you presume assumptions for identification?	No	Further identification remarks	For lipid identification, the Lipidomics Standards Initiative guidelines were followed, employing lipid class-specific fragments to determine the lipid species and molecular lipid species-specific fragments for the annotation of hydrocarbon chains.
Check isomer overlap	Yes		

13) LPE[M+H]+ / Lipid quantification

Quantitative	No	Batch correction	No
Normalization to reference	No	Further quantification remarks	The reported lipid abundances were semi-quantitative, calculated by dividing the analyte response by that of the corresponding internal standard.

14) LPE O[M-H]- / Lipid identification

Lipid class	LPE O	Limit of detection	No
MS Level for identification	MS1, MS2	RT verified by standard	No
Identification level	Species level	Separation of isobaric/isomeric interferece confirmed	No
Polarity mode	Negative	Model for separation prediction	No
Type of negative (precursor)ion	[M-H]-	Additional dimension/techniques	IMS
Fragments for identification		CCS verified by standard	No

Fragment name
-FA1(-H)
GP(153)

Isotope correction at MS1	No	How was/were the additional dimension(s) used?	CCS was used (in combination with other parameters) in the identification of lipids, separation of potential isobaric/isomeric interference.
Isotope correction at MS2	No	Was a model used to predict lipid molecule separation?	No
MS1 verified by standard	No	Lipid Identification Software	Lipid identification was done using MetaboScape 2023b (Bruker Daltonics) utilising an in-house generated retention time and m/z database, rule-based lipid annotation and the MS DIAL MS/MS library LipidBlast (version 68).
MS2 verified by standard	No	Data manipulation	-
Background check at MS1	Yes	Nomenclature for intact lipid molecule	Yes
Background check at MS2	No	Nomenclature for fragment ions	N/A
Did you presume assumptions for identification?	No	Further identification remarks	For lipid identification, the Lipidomics Standards Initiative guidelines were followed, employing lipid class-specific fragments to determine the lipid species and molecular lipid species-specific fragments for the annotation of hydrocarbon chains.
Check isomer overlap	Yes		

14) LPE O[M-H]- / Lipid quantification

Quantitative	No	Batch correction	No
Normalization to reference	No	Further quantification remarks	The reported lipid abundances were semi-quantitative, calculated by dividing the analyte response by that of the corresponding internal standard.

15) LPE O[M+H]+ / Lipid identification

Lipid class	LPE O	Limit of detection	No
MS Level for identification	MS1, MS2	RT verified by standard	No
Identification level	Species level	Separation of isobaric/isomeric interferece confirmed	No
Polarity mode	Positive	Model for separation prediction	No
Type of positive (precursor)ion	[M+H]+	Additional dimension/techniques	IMS
Fragments for identification	Fragment name: -HG(PE,141)	CCS verified by standard	No
Isotope correction at MS1	No	How was/were the additional dimension(s) used?	CCS was used (in combination with other parameters) in the identification of lipids, separation of potential isobaric/isomeric interference.
Isotope correction at MS2	No	Was a model used to predict lipid molecule separation?	No
MS1 verified by standard	No	Lipid Identification Software	Lipid identification was done using MetaboScape 2023b (Bruker Daltonics) utilising an in-house generated retention time and m/z database, rule-based lipid annotation and the MS DIAL MS/MS library LipidBlast (version 68).
MS2 verified by standard	No	Data manipulation	-
Background check at MS1	Yes	Nomenclature for intact lipid molecule	Yes
Background check at MS2	No	Nomenclature for fragment ions	N/A
Did you presume assumptions for identification?	No	Further identification remarks	For lipid identification, the Lipidomics Standards Initiative guidelines were followed, employing lipid class-specific fragments to determine the lipid species and molecular lipid species-specific fragments for the annotation of hydrocarbon chains.
Check isomer overlap	Yes		

15) LPE O[M+H]+ / Lipid quantification

Quantitative	No	Batch correction	No
Normalization to reference	No	Further quantification remarks	The reported lipid abundances were semi-quantitative, calculated by dividing the analyte response by that of the corresponding internal standard.

16) PE[M-H]- / Lipid identification

Lipid class	PE	Limit of detection	No
MS Level for identification	MS1, MS2	RT verified by standard	Yes
Identification level	Molecular species level	Separation of isobaric/isomeric interferece confirmed	No
Polarity mode	Negative	Model for separation prediction	No
Type of negative (precursor)ion	[M-H]-	Additional dimension/techniques	IMS
Fragments for identification		CCS verified by standard	No

Fragment name
HG(PE,196)
GP(153)
FA1(+O)
FA2(+O)
-FA1(+HO)
-FA2(+HO)
-FA1(-H)
-FA2(-H)

Isotope correction at MS1	No	How was/were the additional dimension(s) used?	CCS was used (in combination with other parameters) in the identification of lipids, separation of potential isobaric/isomeric interference.
Isotope correction at MS2	No	Was a model used to predict lipid molecule separation?	No
MS1 verified by standard	Yes	Lipid Identification Software	Lipid identification was done using MetaboScape 2023b (Bruker Daltonics) utilising an in-house generated retention time and m/z database, rule-based lipid annotation and the MS DIAL MS/MS library LipidBlast (version 68).
MS2 verified by standard	No	Data manipulation	-
Background check at MS1	Yes	Nomenclature for intact lipid molecule	No
Background check at MS2	No	Nomenclature for fragment ions	N/A
Did you presume assumptions for identification?	No	Further identification remarks	For lipid identification, the Lipidomics Standards Initiative guidelines were followed, employing lipid class-specific fragments to determine the lipid species and molecular lipid species-specific fragments for the annotation of hydrocarbon chains.
Check isomer overlap	Yes		

16) PE[M-H]- / Lipid quantification

Quantitative	No	Batch correction	No
Normalization to reference	No	Further quantification remarks	The reported lipid abundances were semi-quantitative, calculated by dividing the analyte response by that of the corresponding internal standard.

17) PE[M+H]+ / Lipid identification

Lipid class	PE	Limit of detection	No
MS Level for identification	MS1, MS2	RT verified by standard	Yes
Identification level	Molecular species level	Separation of isobaric/isomeric interferece confirmed	No
Polarity mode	Positive	Model for separation prediction	No
Type of positive (precursor)ion	[M+H]+	Additional dimension/techniques	IMS
Fragments for identification	Fragment name: -HG(PE,141); FA1; FA2	CCS verified by standard	No
Isotope correction at MS1	No	How was/were the additional dimension(s) used?	CCS was used (in combination with other parameters) in the identification of lipids, separation of potential isobaric/isomeric interference.
Isotope correction at MS2	No	Was a model used to predict lipid molecule separation?	No
MS1 verified by standard	Yes	Lipid Identification Software	Lipid identification was done using MetaboScape 2023b (Bruker Daltonics) utilising an in-house generated retention time and m/z database, rule-based lipid annotation and the MS DIAL MS/MS library LipidBlast (version 68).
MS2 verified by standard	No	Data manipulation	-
Background check at MS1	Yes	Nomenclature for intact lipid molecule	No
Background check at MS2	No	Nomenclature for fragment ions	N/A
Did you presume assumptions for identification?	No	Further identification remarks	For lipid identification, the Lipidomics Standards Initiative guidelines were followed, employing lipid class-specific fragments to determine the lipid species and molecular lipid species-specific fragments for the annotation of hydrocarbon chains.
Check isomer overlap	Yes		

17) PE[M+H]+ / Lipid quantification

Quantitative	No	Batch correction	No
Normalization to reference	No	Further quantification remarks	The reported lipid abundances were semi-quantitative, calculated by dividing the analyte response by that of the corresponding internal standard.

18) PE O[M+H]+ / Lipid identification

Lipid class	PE O	Limit of detection	No
MS Level for identification	MS1, MS2	RT verified by standard	No
Identification level	Molecular species level	Separation of isobaric/isomeric interferece confirmed	No
Polarity mode	Positive	Model for separation prediction	No
Type of positive (precursor)ion	[M+H]+	Additional dimension/techniques	IMS
Fragments for identification	Fragment name: -HG(PE,141); FA2+(C3H6O2)	CCS verified by standard	No
Isotope correction at MS1	No	How was/were the additional dimension(s) used?	CCS was used (in combination with other parameters) in the identification of lipids, separation of potential isobaric/isomeric interference.
Isotope correction at MS2	No	Was a model used to predict lipid molecule separation?	No
MS1 verified by standard	No	Lipid Identification Software	Lipid identification was done using MetaboScape 2023b (Bruker Daltonics) utilising an in-house generated retention time and m/z database, rule-based lipid annotation and the MS DIAL MS/MS library LipidBlast (version 68).
MS2 verified by standard	No	Data manipulation	-
Background check at MS1	Yes	Nomenclature for intact lipid molecule	Yes
Background check at MS2	No	Nomenclature for fragment ions	N/A
Did you presume assumptions for identification?	No	Further identification remarks	For lipid identification, the Lipidomics Standards Initiative guidelines were followed, employing lipid class-specific fragments to determine the lipid species and molecular lipid species-specific fragments for the annotation of hydrocarbon chains.
Check isomer overlap	Yes		

18) PE O[M+H]+ / Lipid quantification

Quantitative	No	Batch correction	No
Normalization to reference	No	Further quantification remarks	The reported lipid abundances were semi-quantitative, calculated by dividing the analyte response by that of the corresponding internal standard.

19) PE O[M-H]- / Lipid identification

Lipid class	PE O	Limit of detection	No
MS Level for identification	MS1, MS2	RT verified by standard	No
Identification level	Molecular species level	Separation of isobaric/isomeric interferece confirmed	No
Polarity mode	Negative	Model for separation prediction	No
Type of negative (precursor)ion	[M-H]-	Additional dimension/techniques	IMS
Fragments for identification		CCS verified by standard	No

Fragment name
GP(153)
GP(135)
FA2(+O)
-FA2(+HO)
-FA2(-H)
FA2 -(CO)

Isotope correction at MS1	No	How was/were the additional dimension(s) used?	CCS was used (in combination with other parameters) in the identification of lipids, separation of potential isobaric/isomeric interference.
Isotope correction at MS2	No	Was a model used to predict lipid molecule separation?	No
MS1 verified by standard	No	Lipid Identification Software	Lipid identification was done using MetaboScape 2023b (Bruker Daltonics) utilising an in-house generated retention time and m/z database, rule-based lipid annotation and the MS DIAL MS/MS library LipidBlast (version 68).
MS2 verified by standard	No	Data manipulation	-
Background check at MS1	Yes	Nomenclature for intact lipid molecule	Yes
Background check at MS2	No	Nomenclature for fragment ions	N/A
Did you presume assumptions for identification?	No	Further identification remarks	For lipid identification, the Lipidomics Standards Initiative guidelines were followed, employing lipid class-specific fragments to determine the lipid species and molecular lipid species-specific fragments for the annotation of hydrocarbon chains.
Check isomer overlap	Yes		

19) PE O[M-H]- / Lipid quantification

Quantitative	No	Batch correction	No
Normalization to reference	No	Further quantification remarks	The reported lipid abundances were semi-quantitative, calculated by dividing the analyte response by that of the corresponding internal standard.

20) PI[M-H]- / Lipid identification

Lipid class	PI	Limit of detection	No
MS Level for identification	MS1, MS2	RT verified by standard	Yes
Identification level	Molecular species level	Separation of isobaric/isomeric interferece confirmed	No
Polarity mode	Negative	Model for separation prediction	No
Type of negative (precursor)ion	[M-H]-	Additional dimension/techniques	IMS
Fragments for identification		CCS verified by standard	No

Fragment name
-FA1(-H)
FA1(+O)
-FA1(+HO)
-FA2(-H)
FA2(+O)
-FA2(+HO)
HG(PI,241)

Isotope correction at MS1	No	How was/were the additional dimension(s) used?	CCS was used (in combination with other parameters) in the identification of lipids, separation of potential isobaric/isomeric interference.
Isotope correction at MS2	No	Was a model used to predict lipid molecule separation?	No
MS1 verified by standard	Yes	Lipid Identification Software	Lipid identification was done using MetaboScape 2023b (Bruker Daltonics) utilising an in-house generated retention time and m/z database, rule-based lipid annotation and the MS DIAL MS/MS library LipidBlast (version 68).
MS2 verified by standard	No	Data manipulation	-
Background check at MS1	Yes	Nomenclature for intact lipid molecule	Yes
Background check at MS2	No	Nomenclature for fragment ions	N/A
Did you presume assumptions for identification?	No	Further identification remarks	For lipid identification, the Lipidomics Standards Initiative guidelines were followed, employing lipid class-specific fragments to determine the lipid species and molecular lipid species-specific fragments for the annotation of hydrocarbon chains.
Check isomer overlap	Yes		

20) PI[M-H]- / Lipid quantification

Quantitative	No	Batch correction	No
Normalization to reference	No	Further quantification remarks	The reported lipid abundances were semi-quantitative, calculated by dividing the analyte response by that of the corresponding internal standard.

21) PI[M+NH4]+ / Lipid identification

Lipid class	PI	Limit of detection	No
MS Level for identification	MS1, MS2	RT verified by standard	Yes
Identification level	Species level	Separation of isobaric/isomeric interferece confirmed	No
Polarity mode	Positive	Model for separation prediction	No
Type of positive (precursor)ion	[M+NH4]+	Additional dimension/techniques	IMS
Fragments for identification	Fragment name: -HG(PI,260)	CCS verified by standard	No
Isotope correction at MS1	No	How was/were the additional dimension(s) used?	CCS was used (in combination with other parameters) in the identification of lipids, separation of potential isobaric/isomeric interference.
Isotope correction at MS2	No	Was a model used to predict lipid molecule separation?	No
MS1 verified by standard	Yes	Lipid Identification Software	Lipid identification was done using MetaboScape 2023b (Bruker Daltonics) utilising an in-house generated retention time and m/z database, rule-based lipid annotation and the MS DIAL MS/MS library LipidBlast (version 68).
MS2 verified by standard	No	Data manipulation	-
Background check at MS1	Yes	Nomenclature for intact lipid molecule	Yes
Background check at MS2	No	Nomenclature for fragment ions	N/A
Did you presume assumptions for identification?	No	Further identification remarks	For lipid identification, the Lipidomics Standards Initiative guidelines were followed, employing lipid class-specific fragments to determine the lipid species and molecular lipid species-specific fragments for the annotation of hydrocarbon chains.
Check isomer overlap	Yes		

21) PI[M+NH4]+ / Lipid quantification

Quantitative	No	Batch correction	No
Normalization to reference	No	Further quantification remarks	The reported lipid abundances were semi-quantitative, calculated by dividing the analyte response by that of the corresponding internal standard.

22) PS[M-H]- / Lipid identification

Lipid class	PS	Limit of detection	No
MS Level for identification	MS1, MS2	RT verified by standard	Yes
Identification level	Molecular species level	Separation of isobaric/isomeric interferece confirmed	No
Polarity mode	Negative	Model for separation prediction	No
Type of negative (precursor)ion	[M-H]-	Additional dimension/techniques	IMS
Fragments for identification		CCS verified by standard	No

Fragment name
-FA1(-H)-(C3H5NO2)
FA1(+O)
-FA1(+HO)-(C3H5NO2)
-FA2(-H)-(C3H5NO2)
FA2(+O)
-FA2(+HO)-(C3H5NO2)
-(C3H5NO2,87)

Isotope correction at MS1	No	How was/were the additional dimension(s) used?	CCS was used (in combination with other parameters) in the identification of lipids, separation of potential isobaric/isomeric interference.
Isotope correction at MS2	No	Was a model used to predict lipid molecule separation?	No
MS1 verified by standard	Yes	Lipid Identification Software	Lipid identification was done using MetaboScape 2023b (Bruker Daltonics) utilising an in-house generated retention time and m/z database, rule-based lipid annotation and the MS DIAL MS/MS library LipidBlast (version 68).
MS2 verified by standard	No	Data manipulation	-
Background check at MS1	Yes	Nomenclature for intact lipid molecule	Yes
Background check at MS2	No	Nomenclature for fragment ions	N/A
Did you presume assumptions for identification?	No	Further identification remarks	For lipid identification, the Lipidomics Standards Initiative guidelines were followed, employing lipid class-specific fragments to determine the lipid species and molecular lipid species-specific fragments for the annotation of hydrocarbon chains.
Check isomer overlap	Yes		

22) PS[M-H]- / Lipid quantification

Quantitative	No	Batch correction	No
Normalization to reference	No	Further quantification remarks	The reported lipid abundances were semi-quantitative, calculated by dividing the analyte response by that of the corresponding internal standard.

23) PS[M+H]+ / Lipid identification

Lipid class	PS	Limit of detection	No
MS Level for identification	MS1, MS2	RT verified by standard	Yes
Identification level	Molecular species level	Separation of isobaric/isomeric interferece confirmed	No
Polarity mode	Positive	Model for separation prediction	No
Type of positive (precursor)ion	[M+H]+	Additional dimension/techniques	IMS
Fragments for identification	Fragment name: -HG(PS,185)	CCS verified by standard	No
Isotope correction at MS1	No	How was/were the additional dimension(s) used?	CCS was used (in combination with other parameters) in the identification of lipids, separation of potential isobaric/isomeric interference.
Isotope correction at MS2	No	Was a model used to predict lipid molecule separation?	No
MS1 verified by standard	Yes	Lipid Identification Software	Lipid identification was done using MetaboScape 2023b (Bruker Daltonics) utilising an in-house generated retention time and m/z database, rule-based lipid annotation and the MS DIAL MS/MS library LipidBlast (version 68).
MS2 verified by standard	Yes	Data manipulation	-
Background check at MS1	Yes	Nomenclature for intact lipid molecule	Yes
Background check at MS2	No	Nomenclature for fragment ions	N/A
Did you presume assumptions for identification?	No	Further identification remarks	For lipid identification, the Lipidomics Standards Initiative guidelines were followed, employing lipid class-specific fragments to determine the lipid species and molecular lipid species-specific fragments for the annotation of hydrocarbon chains.
Check isomer overlap	Yes		

23) PS[M+H]+ / Lipid quantification

Quantitative	No	Batch correction	No
Normalization to reference	No	Further quantification remarks	The reported lipid abundances were semi-quantitative, calculated by dividing the analyte response by that of the corresponding internal standard.

24) SM[M+H]+ / Lipid identification

Lipid class	SM	Limit of detection	No
MS Level for identification	MS1, MS2	RT verified by standard	Yes
Identification level	Molecular species level	Separation of isobaric/isomeric interferece confirmed	No
Polarity mode	Positive	Model for separation prediction	No
Type of positive (precursor)ion	[M+H]+	Additional dimension/techniques	IMS
Fragments for identification	Fragment name: HG(PC,184); LCB(-H3O2)	CCS verified by standard	No
Isotope correction at MS1	No	How was/were the additional dimension(s) used?	CCS was used (in combination with other parameters) in the identification of lipids, separation of potential isobaric/isomeric interference.
Isotope correction at MS2	No	Was a model used to predict lipid molecule separation?	No
MS1 verified by standard	Yes	Lipid Identification Software	Lipid identification was done using MetaboScape 2023b (Bruker Daltonics) utilizing an in-house generated retention time and m/z database, rule-based lipid annotation and the MS DIAL MS/MS library LipidBlast (version 68).
MS2 verified by standard	No	Data manipulation	-
Background check at MS1	Yes	Nomenclature for intact lipid molecule	Yes
Background check at MS2	No	Nomenclature for fragment ions	N/A
Did you presume assumptions for identification?	No	Further identification remarks	For lipid identification, the Lipidomics Standards Initiative guidelines were followed, employing lipid class-specific fragments to determine the lipid species and molecular lipid species-specific fragments for the annotation of hydrocarbon chains.
Check isomer overlap	Yes		

24) SM[M+H]+ / Lipid quantification

Quantitative	No	Batch correction	No
Normalization to reference	No	Further quantification remarks	The reported lipid abundances were semi-quantitative, calculated by dividing the analyte response by that of the corresponding internal standard.

25) TG O[M+NH4]+ / Lipid identification

Lipid class	TG O	Limit of detection	No
MS Level for identification	MS1, MS2	RT verified by standard	No
Identification level	Species level	Separation of isobaric/isomeric interferece confirmed	No
Polarity mode	Positive	Model for separation prediction	Yes
Type of positive (precursor)ion	[M+NH4]+	Additional dimension/techniques	IMS
Fragments for identification		CCS verified by standard	Yes

Fragment name
-FA(+HO) -TG(17)
-TG(17)

Isotope correction at MS1	No	How was/were the additional dimension(s) used?	CCS was used (in combination with other parameters) in the identification of lipids, separation of potential isobaric/isomeric interference.
Isotope correction at MS2	No	Was a model used to predict lipid molecule separation?	No
MS1 verified by standard	No	Lipid Identification Software	Lipid identification was done using MetaboScape 2023b (Bruker Daltonics) utilising an in-house generated retention time and m/z database, rule-based lipid annotation and the MS DIAL MS/MS library LipidBlast (version 68).
MS2 verified by standard	No	Data manipulation	-
Background check at MS1	Yes	Nomenclature for intact lipid molecule	No
Background check at MS2	No	Nomenclature for fragment ions	N/A
Did you presume assumptions for identification?	No	Further identification remarks	For lipid identification, the Lipidomics Standards Initiative guidelines were followed, employing lipid class-specific fragments to determine the lipid species and molecular lipid species-specific fragments for the annotation of hydrocarbon chains.
Check isomer overlap	No		

25) TG O[M+NH4]+ / Lipid quantification

Quantitative	No	Batch correction	No
Normalization to reference	No	Further quantification remarks	The reported lipid abundances were semi-quantitative, calculated by dividing the analyte response by that of the corresponding internal standard.

26) LPI[M-H]- / Lipid identification

Lipid class	LPI	Limit of detection	No
MS Level for identification	MS1, MS2	RT verified by standard	No
Identification level	Molecular species level	Separation of isobaric/isomeric interferece confirmed	No
Polarity mode	Negative	Model for separation prediction	No
Type of negative (precursor)ion	[M-H]-	Additional dimension/techniques	IMS
Fragments for identification	Fragment name: GP(153); HG(PI,241); -FA1(+HO); FA1(+O)	CCS verified by standard	No
Isotope correction at MS1	No	How was/were the additional dimension(s) used?	CCS was used (in combination with other parameters) in the identification of lipids, separation of potential isobaric/isomeric interference.
Isotope correction at MS2	No	Was a model used to predict lipid molecule separation?	No
MS1 verified by standard	Yes	Lipid Identification Software	Lipid identification was done using MetaboScape 2023b (Bruker Daltonics) utilizing an in-house generated retention time and m/z database, rule-based lipid annotation and the MS DIAL MS/MS library LipidBlast (version 68).
MS2 verified by standard	No	Data manipulation	-
Background check at MS1	Yes	Nomenclature for intact lipid molecule	Yes
Background check at MS2	No	Nomenclature for fragment ions	N/A
Did you presume assumptions for identification?	No	Further identification remarks	For lipid identification, the Lipidomics Standards Initiative guidelines were followed, employing lipid class-specific fragments to determine the lipid species and molecular lipid species-specific fragments for the annotation of hydrocarbon chains.
Check isomer overlap	Yes		

26) LPI[M-H]- / Lipid quantification

Quantitative	No	Batch correction	No
Normalization to reference	No	Further quantification remarks	The reported lipid abundances were semi-quantitative, calculated by dividing the analyte response by that of the corresponding internal standard.

27) LPS[M-H]- / Lipid identification

Lipid class	LPS	Limit of detection	No
MS Level for identification	MS1, MS2	RT verified by standard	No
Identification level	Molecular species level	Separation of isobaric/isomeric interferece confirmed	No
Polarity mode	Negative	Model for separation prediction	No
Type of negative (precursor)ion	[M-H]-	Additional dimension/techniques	IMS
Fragments for identification		CCS verified by standard	No

Fragment name
-(C3H5NO2,87)
FA1(+O)
GP(153)

Isotope correction at MS1	No	How was/were the additional dimension(s) used?	CCS was used (in combination with other parameters) in the identification of lipids, separation of potential isobaric/isomeric interference.
Isotope correction at MS2	No	Was a model used to predict lipid molecule separation?	No
MS1 verified by standard	Yes	Lipid Identification Software	Lipid identification was done using MetaboScape 2023b (Bruker Daltonics) utilizing an in-house generated retention time and m/z database, rule-based lipid annotation and the MS DIAL MS/MS library LipidBlast (version 68).
MS2 verified by standard	No	Data manipulation	-
Background check at MS1	Yes	Nomenclature for intact lipid molecule	Yes
Background check at MS2	No	Nomenclature for fragment ions	N/A
Did you presume assumptions for identification?	No	Further identification remarks	For lipid identification, the Lipidomics Standards Initiative guidelines were followed, employing lipid class-specific fragments to determine the lipid species and molecular lipid species-specific fragments for the annotation of hydrocarbon chains.
Check isomer overlap	Yes		

27) LPS[M-H]- / Lipid quantification

Quantitative	No	Batch correction	No
Normalization to reference	No	Further quantification remarks	The reported lipid abundances were semi-quantitative, calculated by dividing the analyte response by that of the corresponding internal standard.

28) LPS[M+H]+ / Lipid identification

Lipid class	LPS	RT verified by standard	No
MS Level for identification	MS1	Separation of isobaric/isomeric interferece confirmed	Yes
Identification level	Species level	Model for separation prediction	No
Polarity mode	Positive	Additional dimension/techniques	IMS
Type of positive (precursor)ion	[M+H]+	CCS verified by standard	No
Isotope correction at MS1	No	How was/were the additional dimension(s) used?	CCS was used (in combination with other parameters) in the identification of lipids, separation of potential isobaric/isomeric interference.
MS1 verified by standard	No	Was a model used to predict lipid molecule separation?	No
Background check at MS1	Yes	Lipid Identification Software	Lipid identification was done using MetaboScape 2023b (Bruker Daltonics) utilizing an in-house generated retention time and m/z database, rule-based lipid annotation and the MS DIAL MS/MS library LipidBlast (version 68).
Did you presume assumptions for identification?	No	Data manipulation	-
Check isomer overlap	Yes	Nomenclature for intact lipid molecule	Yes
Limit of detection	No	Further identification remarks	For lipid identification, the Lipidomics Standards Initiative guidelines were followed, employing lipid class-specific fragments to determine the lipid species and molecular lipid species-specific fragments for the annotation of hydrocarbon chains.

28) LPS[M+H]+ / Lipid quantification

Quantitative	No	Batch correction	No
Normalization to reference	No	Further quantification remarks	The reported lipid abundances were semi-quantitative, calculated by dividing the analyte response by that of the corresponding internal standard.

29) SLBPA[M-H]- / Lipid identification

Lipid class	SLBPA	RT verified by standard	No
MS Level for identification	MS1	Separation of isobaric/isomeric interferece confirmed	No
Identification level	Species level	Model for separation prediction	No
Polarity mode	Negative	Additional dimension/techniques	IMS
Type of negative (precursor)ion	[M-H]-	CCS verified by standard	No
Isotope correction at MS1	No	How was/were the additional dimension(s) used?	CCS was used (in combination with other parameters) in the identification of lipids, separation of potential isobaric/isomeric interference.
MS1 verified by standard	No	Was a model used to predict lipid molecule separation?	No
Background check at MS1	No	Lipid Identification Software	Lipid identification was done using MetaboScape 2023b (Bruker Daltonics) utilising an in-house generated retention time and m/z database, rule-based lipid annotation and the MS DIAL MS/MS library LipidBlast (version 68).
Did you presume assumptions for identification?	No	Data manipulation	-
Check isomer overlap	No	Nomenclature for intact lipid molecule	No
Limit of detection	No	Further identification remarks	For lipid identification, the Lipidomics Standards Initiative guidelines were followed, employing lipid class-specific fragments to determine the lipid species and molecular lipid species-specific fragments for the annotation of hydrocarbon chains.

29) SLBPA[M-H]- / Lipid quantification

Quantitative	No	Batch correction	No
Normalization to reference	No	Further quantification remarks	The reported lipid abundances were semi-quantitative, calculated by dividing the analyte response by that of the corresponding internal standard.
